# Visual perception and dissociation during Mirror Gazing Test in patients with anorexia nervosa: a preliminary study

**DOI:** 10.1007/s40519-020-00977-6

**Published:** 2020-08-05

**Authors:** Benedetta Demartini, Veronica Nisticò, Roberta Tedesco, Andrea Marzorati, Roberta Ferrucci, Alberto Priori, Orsola Gambini, Giovanni B. Caputo

**Affiliations:** 1 Dipartimento di Scienze della Salute, Università degli Studi di Milano, A.O. San Paolo, via A. di Rudinì, 8, 20142 Milan, Italy; 2 “Aldo Ravelli” Research Center for Neurotechnology and Experimental Brain Therapeutics, University of Milan, Milan, Italy; 3 Unità di Psichiatria II, A.O. San Paolo, ASST Santi Paolo e Carlo, Milan, Italy; 4 III Clinica Neurologica, A.O. San Paolo, ASST Santi Paolo e Carlo, Milan, Italy; 5 DISTUM, University of Urbino, Urbino, Italy

**Keywords:** Anorexia nervosa, Eating disorders, Mirror Gazing Test, Dissociation, Body image

## Abstract

**Purpose:**

It has been widely shown that dissociative features might play a fundamental role in producing body image distortions in patients affected by eating disorders. Here, we hypothesize that the Mirror Gazing Test (MGT), a task consisting in mirror exposure in a condition of sensory deprivation, would elicit dissociative symptoms in a group of patients with anorexia nervosa (AN).

**Methods:**

Fourteen patients with AN and fourteen healthy controls (HC) underwent a 10 min MGT and completed the Strange Face Questionnaire and a short version of the Clinician-Administered Dissociative States Scale, along with a psychological assessment for eating disorders psychopathology, anxiety and depression.

**Results:**

AN patients reported a higher number of strange-face apparitions and dissociative sensations than HC during the MGT. Dissociative identity (compartmentalization of two or more identities) and depersonalization (detachment of bodily-self) were much higher in patients with AN than in HC. These findings were correlated with body dissatisfaction and disruption in interoceptive awareness.

**Conclusion:**

Dissociation and body image dysfunction are strongly connected in the pathophysiology of anorexia nervosa. Future research should investigate the same aspects in other psychiatric conditions characterized by body image distortions, such as Body Dysmorphic Disorder.

**Level of evidence:**

I, Experimental studies.

## Introduction

Body image is commonly defined as “the picture of our own body which we form in our mind” [1], as it appears when observed in a third-person perspective. It can be considered a multidimensional construct, implying both bottom-up (visual and proprioceptive information) and top–down facets (such as memory, mood states, affects, attitudes and beliefs concerning appearance). With respect to body image disruption, two facets are traditionally distinguished: perceptual distortion and body dissatisfaction [2, 3]. Perceptual distortion is defined as the inability to accurately perceive one’s body size, and it is usually measured through visual tasks; body dissatisfaction represents the extent to which people are satisfied with their bodies’ size and shape and it is commonly measured by rating scales and questionnaires [4, 5]. Body image disruptions emerge in different neuropsychiatric disorders and conditions, such as Eating Disorders (ED) and Body Dysmorphic Disorder (BDD). Signs of body image disruptions in ED frequently emerge during self-gazing: it is reported that when looking at their own body, women with anorexia nervosa (AN) and bulimia nervosa attended more to their self-identified most unattractive body part than to their self-identified most attractive body part, contrary to healthy controls (HC) [6]. Patients with ED, moreover, tend to present stronger negative affect than HC during the course of a mirror exposure [7–9]. Following this framework, the Allocentric Lock Theory tried to explain the body image disruption seen in patients with ED: ED might be explained as being locked on a negative body image (e.g., my body is fat). This might be the result of an updated percept stored in the short-term memory (egocentric), which does not reach the long-term memory to allow for a change in body image as perceived from the outside (allocentric). Thus, the ability to encode and use visual information from the environment is impaired. Consequently, the egocentric sensory inputs are not able to update the contents of the allocentric representation of the body, and so the person is locked into this distorted perception [10, 11]. The distortion of body image in ED might also be linked to perceptual biases, such as the visual body image adaptation (due to a pre-existing, long-lasting adaptation to thin body shapes in ED) [12] or the so-called serial dependence (a phenomenon occurring when errors in perceptual judgements are consistent with the assimilation of features of a previously viewed stimulus with the current stimulus) [13]. Moreover, from a neurobiological perspective, it has been hypothesized that body image distortion in patients with AN could derive from abnormal functioning of the insula, an area of the brain that integrates interoceptive awareness, involving the sensory process of receiving, accessing and appraising internal bodily signals [14, 15]. In particular, the activity of the anterior insula, which may represent motivational tendencies, but is also part of the fear and emotional network, has been found to be diminished in anorexic patients [16].

In the last decades, the hypothesis that dissociation might play a fundamental role in body image distortion, has been explored. It has been even hypothesized that body image distortion is a kind of psychoform dissociation, where the patient perceives his/her body in dimensions that are not real and idealises and fantasises about a body that usually does not match the real one [17]. The presence of dissociative symptoms in ED is now well-established and is not limited to AN: dissociative amnesia, depersonalization and derealisation are common characteristics of binge-eating episodes [18]; dissociative tendencies represent important mediators between childhood abuse and all subtypes of eating disturbances, including subclinical ones [19]; moreover, patients with AN show significantly higher rates of dissociative features than HC; finally, it seems that patients with AN with a binge-eating/purging subtype present higher levels of dissociative symptoms than patients affected by AN restrictive subtype and than HC when assessed via self-report clinical questionnaires [20]. However, despite the link between body image disruption and dissociative symptoms in ED, other possible correlates of dissociation, such as traumatic experiences, have been considered: Van IJzendoorn and Schuengel, in their meta-analysis, concluded that, in some patients, childhood abuse experiences might lead to an ED as well as to a dissociative disorder [21]. Moreover, it has been hypothesized that the heightened attention towards his/her most unattractive body part might be a reflex of patient’s selective attention towards threats due to the presence of dissociative features [22, 23]. Furthermore, Mussap and Salton [24] believe that dissociative processes, known to determine a disconnection of one’s sense of self, might be responsible for the variability in body size estimates. This hypothesis is further sustained by the study of Fuller-Tyszkiewicz et al. [23], which found a significative correlation between body image perception (assessed via a computer-based task) and dissociative symptoms, investigated via self-report questionnaires such as the Somatoform Dissociation Questionnaire in a non-clinical sample (student sample). This finding was replicated also in clinical samples: Beato and colleagues found, through the administration of self-report questionnaires, dissociation to be associated to the degree of dissatisfaction towards their body in a sample of patients with ED [25]. Nevertheless, one of the main limitations of these previous studies was the lack of an objective assessment of dissociation and its subcomponents, which were evaluated only through self-report scales and not by experimental tasks.

Here we implemented a specific task, the Mirror Gazing Test (MGT) [26, 27], to assess dissociative symptoms, elicited by mirror exposure, in a group of AN patients. Specifically, the participant is asked to steadily look at his/her face image reflected in a mirror, in a dimly lit room for ten minutes. It has been reported that, during the MGT, several visual illusions occur (such as deformations of one’s own face, a relative’s face with some changed features or an unknown person’s face; an archetypal or an animal face), often accompanied by dissociative phenomena (i.e., the feeling that the reflected face did not belong to themselves anymore plus the feeling of being watched by “the strange other” from within or beyond the mirror) [28]. These sensations physiologically occur in healthy subjects [26, 28], but seem to be strongly connected with psychopathological features, since they emerge more frequently in schizophrenic individuals [29] and in patients with functional neurological disorders [30] and less frequently in depressed patients [31] when compared to HC. It has been hypothesized that strange-face apparitions might emerge during MGT, even in healthy subjects because of a disconnection of two facets of representations that are usually recruited together and allow self-recognition in the mirror: the one’s own embodied self-representation (perceived through somesthetic, kinesthetic, and proprioceptive signals) and the visual representation of one’s own face left–right reversed, as it usually appears in the mirror. During this unbinding process, the so-called “strange Other” (i.e., an alter- identity) appears in the mirror, watching the embodied self, thus producing a perception of dissociated self-identity [26]. Moreover, Shin et al. recently provided experimental evidences, with the use of the MGT on a sample of healthy individuals, for the short-term alleviation (i.e., emotional numbing) of negative affect during acute dissociative states induced by the exposure to the MGT, which may serve as a coping mechanism for some individuals [31]. It is important to mention that self-identity dissociation during MGT is different from the kind of dissociation found in patients suffering from dissociative identity disorder. In fact, during the MGT participants maintain embodied consciousness of themselves, while in patients suffering from dissociative identity disorder consciousness is lost and dissociative identity remains unconscious to the normal personality.

To summarise, the current study tested the presence of dissociative symptoms, elicited by mirror exposure via the MGT, in a group of anorexic patients. Given previous findings showing that individuals with AN are characterized by body image disruption, that body image disruption is strictly linked to dissociative features and that dissociation, assessed by self-report questionnaires, is a common phenomenon in anorexic patients, we would expect patients with AN to present an increased amount of strange face apparitions and dissociative sensations, elicited by the mirror exposure during the MGT when compared to HC. Moreover, in the light of the well-established involvement of the insula function in the pathogenesis of AN, in this study we also tested the hypothesis that the increased amount of dissociative symptoms would be associated not only with body image disruptions, but also with interoceptive awareness.

## Methods

### Participants

Fourteen out-patients with a diagnosis of AN were recruited from the Eating Disorder Outpatient Clinic of San Paolo General Hospital in Milan, Italy. Patients were enrolled in the study as soon as they came for the first evaluation and they did not previously receive any kind of treatment. They were compared with 14 HC, recruited from staff members, their friends and relatives, through advertisement and word of mouth. The HC “healthy state” was determined through a specifically designed anamnestic interview, including questions on current medical history and the administration of the clinical interview Structured Clinical Interview for DSM-5 (SCID-5) [33]. Although some of the HC were staff members, none of them were aware of the study aim and protocol. Diagnosis of AN was made according to DSM-5 diagnostic criteria. Other psychiatric, neurological or medical disorders were excluded through a complete anamnestic questionnaire and a clinical interview (SCID-5) [33]). Participants of the two groups were Caucasian. The Body Mass Index (BMI) was calculated for every participant, with the following formula: weight/height^2^ kg/m^2^.

Exclusion criteria were as follows: (i) age below 18 years and above 50 years; (ii) inability to understand the aim and the steps of the project due to cognitive impairment; (iii) any other serious psychiatric, neurological or medical illnesses; (iv) previous diagnosis of other ED (which might represent a confounding factor) for the patients with AN and of any ED for the HC; (v) a BMI > 18 for the patients with AN, and a BMI < 18 for the HC.

To take part in the study, all the participants signed an informed consent form with detailed study information. Every participant had the opportunity to ask for clarification and explanation during each stage of the study and was free to interrupt and leave the experiment at any moment. The study was approved and registered by the ethics committee of ASST Santi Paolo e Carlo, Milan, Italy. The experiment was conducted in accordance with the Declaration of Helsinki.

### Experimental protocol

MGT was conducted in a 5 m × 5 m room, with obscured windows. A mirror (0.5 m × 0.5 m) was mounted on a tripod in the centre of the room. Each subject seated in front of the mirror at a distance of 0.4 m; the only source of light of the whole room (a halogen light bulb, 20 W) was placed on the floor 1.2 m behind the subject (out of the participant’s visual field and of the mirror reflection). Illumination of the face (‘incident light’) was about 1 lx (measured with TES-1330 A luxmeter). Each participant was instructed to keep staring into his/her own eyes for ten minutes. To double check that participants correctly performed the task, the experiment was seated in the room in a position, where he could see the participant, but where the participant could not see him. For further details about the procedure see Caputo et al. [29]. At the end of the session, participants completed the Strange Face Questionnaire (SFQ), an ad-hoc questionnaire assessing anomalous sensation and anomalous perceptions they had looking in the mirror ([34]; for psychometric properties see [27]) and a short Italian version of the Clinician-Administered Dissociative States Scale (CADSS) [35]. The CADSS items were adapted to past-tense verbal sentences from the present tense of the original version.

The Strange Face Questionnaire is composed by 28 items describing possible sensations or perceptions occurring during the MGT, to be replied on a 5-point Likert scale (where 0 meant “I never experienced this sensation” and 4 meant “I experienced this sensation almost always”). Item 19 is a control item: thus, the questionnaire was considered valid only if the answer was 0, never. A total score, ranging from 0 to 108, was calculated by summing the answer to the other 27 items responses. The scoring procedure was as follows: firstly, number of answers “never” and number of answers ranging from 1 to 4 were counted, as an index of how many different apparitions and sensations occurred during the MGT (range: 0–27); secondly, a total score, ranging from 0 to 108, was calculated. Finally, three subscales have been calculated and analysed: (i) Derealisation (summing items: 1, 4, 5, 6, 8, 10, 11, 16; total score ranging from 0 to 32; example: “Did you seem to see that the face was deformed or some features of the face were deformed?”); (ii) Depersonalization (summing items: 14, 17, 18, 20, 22, 23, 24; total score ranging from 0 to 28; example: “Did you seem to perceive a "presence" of something that does not physically exist?”); (iii) Dissociative Identity/Compartmentalization (summing items: 2, 7, 9, 12, 13, 21, 25, 26, 27; total score ranging from 0 to 36; example: “Did you seem to recognize another personality that you would not have expected?”). The three subscales of SFQ present good levels of reliability and validity: SFQ-Derealisation (α = 0.73), SFQ-Depersonalization (α = 0.32), and SFQ-Dissociative Identity (α = 0.61) [27].

The short version of the Clinician-Administered Dissociative States Scale is composed by 19 items, to be replied on a 5-point Likert scale (0 being “never” and 4 being “almost always”). An overall total score, ranging from 0 to 76, was calculated by summing each single item’ response; moreover, the three following subscales were calculated and analysed: (i) Derealisation (summing items: 1, 2, 8, 9, 10, 11, 12, 13, 16, 17, 18, 19; total score ranging from 0 to 48; example: “did it seem like things were unreal, as if you were in a dream?”); (ii) Depersonalization (summing items: 3, 4, 5, 6, 7; total score ranging from 0 to 20; example: “did it seem as if you were looking at things from outside your own body?”); (iii) Dissociative Amnesia (summing items: 14, 15; total score ranging from 0 to 8; example: “did it seem like you were wandering with your own thoughts and/or losing track of what was happening?”). The three subscales of CADSS present good levels of reliability and validity: CADSS-Derealisation (α = 0.64), CADSS-Depersonalization (α = 0.62), and CADSS-Dissociative Amnesia (α = 0.33) [27]. Furthermore, participants completed the Eating Disorder Inventory-2 (EDI-2), assessing alimentary habits and clusters of symptoms deemed relevant for the understanding and treatment of ED. The eight original subscales of the EDI-2 present high levels of reliability and validity (all α > 0.80). In particular, we selected and analysed the two subscales Body Dissatisfaction (α = 0.91) and Interoceptive Awareness (α = 0.84) [36, 37].

At the very end of the experiment we allowed the participants ten minutes to relax and ask questions before leaving the setting.

### Data analysis

Data were analysed through the software SPSS (Statistical Package for Social Science), Version 25. Significance levels were set at *p* < 0.05, two tailed. We did not have any missing data. First, reliability analysis was run to assess the internal consistency of each scale in our sample. Second, given the small sample size, non-parametric tests have been run. Specifically, Mann–Whitney *U* test was used to assess the differences between the two groups at the MGT questionnaires and at the psychological assessment. Categorical variables were analysed via Pearson Chi Square (*χ*^2^) test. To test the hypothesis that MGT-elicited dissociation would be associated with body image disruption and interoceptive awareness, a series of multiple linear regressions analysis was run, with the SFQ and the CADSS as dependent variables (DV) and with the following independent variables (IV): (i) the dichotomic variable Group, where 0 = HC and 1 = patients with AN; (ii) BMI; (iii) the two EDI-2 subscales Body Dissatisfaction and Interoceptive Awareness. Given the involvement of 5 variables (1 DV, 4 IV), the lower limit of “two subjects per variable” for linear regression has been respected [38]; nonetheless, both *R*^2^ and adjusted *R*^2^ were reported.

Additionally, within the AN group only, Spearman’s correlational analysis was run between the continuous variable Years of illness and the MGT variables to investigate possible covariations.

## Results

### Reliability analysis

Reliability analysis (Cronbach’s α) showed that the internal consistency within our sample of the MGT questionnaires was as follows: (i) SFQ Total Score: α = 0.787; (ii) SFQ Derealisation: α = 0.672; (iii) SFQ Depersonalization: α = 0.491; (iv) SFQ Dissociative Identity/Compartmentalization: α  = 0.730; (v) CADSS Total Score: α  = 0.894; (vi) CADSS Derealisation: α = 0.758; (vii) CADSS Depersonalization; α = 0.828; (viii) CADSS Dissociative Amnesia: α = 0.444. Moreover, the internal consistency within our sample of the EDI-2 Total score was high (α = 0.973), as well as of both the EDI-2 subscales: (i) Body Dissatisfaction: α = 0.891; (ii) Interoceptive Awareness: α = 0.895.

### Demographic data

AN and HC samples were sex- (*χ* (1) = 3.360, *p* = 0.067) and age- (*U* (28) = 72, *p* = 0.246) matched. Out of 14 patients recruited, 12 had a diagnosis of restrictive subtype AN and 2 of purging subtype AN. Disease duration was 11.73 (± 11.17) years. Patients with AN and HC did not differ with respect to family psychiatric history (*χ* (1) = 0.622, *p* = 0.430). Analysis showed that, not only the current average BMI was different between the two groups (*U* (28) = 0, *p* < 0.001), but also the highest (*U* (28) = 29, *p* < 0.001) and the lowest BMI ever reached (*U* (28) = 0, *p* < 0.001). Demographic, experimental and psychological values are reported in Table 1.

**Table 1:** Values for demographic, psychometric and experimental variables

	Anorexic patients	Healthy controls	*p*
Sex (M/F)	0/14	3/11	0.067
Age, year (SD)	28.3 (10.7)	31.7 (9.8)	0.246
Family psychiatric history (Y/N)	6/8	4/10	0.430
Disease duration, mean years (SD)	11.73 (11.17)	N/A	N/A
Current BMI, mean (SD)	15.5 (1.3)	22.7 (3.4)	** < 0.001**
Lowest BMI, mean (SD)	13.3 (1.6)	20 (2.2)	** < 0.001**
Highest BMI, mean (SD)	20.2 (2.2)	24.575 (4)	** < 0.001**
SFQ—total score, mean (SD)	24.4 (10.1)	11.4 (7)	**0.001**
SFQ—*n*° answer YES, mean (SD)	11.8 (4.7)	6 (3.7)	**0.001**
SFQ—derealization, mean (SD)	7.1 (4.5)	3.7 (3.2)	0.085
SFQ—depersonalization, mean (SD)	4.3 (3.2)	2.8 (2.9)	**0.05**
SFQ—dissociative identity/compartmentalization, mean (SD)	6.9 (4.7)	0.8 (1.2)	** < 0.001**
CADSS—total score, mean (SD)	26 (11.5)	9.6 (10.2)	**0.001**
CADSS—derealization, mean (SD)	14.8 (6.7)	6.6 (5.8)	**0.004**
CADSS—depersonalization, mean (SD)	8.4 (4.5)	1.9 (3.4)	** < 0.001**
CADSS—dissociative amnesia, mean (SD)	2.9 (2.2)	1.1 (1.7)	**0.024**
EDI-2—total score	21.3 (17.4)	98.8 (41.1)	** < 0.001**
EDI-2—body dissatisfaction, mean (SD)	15.4 (7.1)	3.9 (4.3)	** < 0.001**
EDI-2—interoceptive awareness, mean (SD)	10.4 (7.2)	0.8 (1.5)	** < 0.001**

*SD* Standard deviation, *M* Male, *F* Female, *Y* Yes, *N* No, *BMI* Body Mass Index, *SFQ* Strange Face Questionnaire, *CADSS* Clinician-Administered Dissociative States Scale, *EDI-2* Eating Disorder Inventory—2

Bold values are significant values (*p* < 0.05)

### Mirror Gazing Test (MGT)

The number of times participants had misperceptions by looking in the mirror, as assessed via the SFQ, differed between the two groups (*U* (28) = 166, *p* = 0.001) with AN patients having more strange-face anomalous perceptions/experiences than HC. Patients with AN reported higher values than HC at the SFQ total score (*U* (28) = 166, *p* = 0.001); a significant difference between the two groups emerged at the SFQ subscale Dissociative Identity/Compartmentalization (*U* (28) = 179.5, *p* < 0.001), with AN scoring higher than HC; a trend towards significance emerged also at the SFQ subscale Depersonalization (*U* (28) = 141, *p* = 0.050), again with AN scoring higher than HC, but no difference was detected at the Derealisation SFQ subscale (*U* (28) = 135.5, *p* = 0.085). Moreover, AN patients scored higher than HC at the CADSS total score (*U* (28) = 169.5, *p* < 0.001) and at all CADSS subscales: Derealisation: *U* (28) = 159, *p* = 0.004; Depersonalisation: *U* (28) = 177, *p* < 0.001; Dissociative Amnesia: *U* (28) = 147, *p* = 0.024. Finally, patients with AN showed significantly higher values than HC at the EDI-2 Total Score (*U* (28) = 193, *p* < 0.001) and at both the EDI-2 subscales: (i) Body Dissatisfaction: *U* (28) = 177, *p* < 0.001; (ii) Interoceptive Awareness: *U* (28) = 193, *p* < 0.001.

### Regression and correlational analysis

In a series of multiple regressions, neither the variable Group nor the variable BMI was associated with any of the MGT variables. The EDI-2 subscale Body Dissatisfaction was positively correlated to the SFQ subscale Derealization (*b* = 0.299, *t* = 2.323, *p* = 0.029) and to the CADSS subscale Dissociative Amnesia (*b* = 0.197, *t* = 3.297, *p* = 0.003). Additionally, a trend towards significance emerged for the relationship between the dependent variable Body Dissatisfaction and the independent variable SFQ Total Score (*b* = 0.582, *t* = 2.03, *p* = 0.054). Finally, the EDI-2 subscale Interoceptive Awareness was positively correlated to the CADSS Total Score (*b* = 0.892, *t* = 2.415, *p* = 0.024), and to the CADSS subscales Depersonalization (*b* = 0.37, *t* = 2.674, *p* = 0.014) and Dissociative Amnesia (*b* = 0.197, *t* = 3.297, *p* = 0.003). Further values are shown in Table 2. Duration of disease was not correlated with any of the MGT variables (all *p* > 0.05).

**Table 2:** Multiple linear regression with the MGT variables as dependent variables and Group, BMI and the EDI-2 subscales as predictors

	SFQ tot					SFQ der					SFQ dep					SFQ D.I				
	*b*	*t*	*p*	*R*^2^	Adj *R*^2^	*b*	*t*	*p*	*R*^2^	Adj *R*^2^	*b*	*t*	*p*	*R*^2^	Adj *R*^2^	*b*	*t*	*p*	*R*^2^	Adj *R*^2^
Intercept	13.948	0.944	0.355	0.499	0.412	2.9	0.431	0.670	0.336	0.220	1.898	0.332	0.743	0.077	− 0.084	3.177	0.509	0.616	0.500	0.413
Group	2.299	0.325	0.748			− 0.4	− 0.131	0.897			1.877	0.685	0.500			2.882	0.965	0.345		
BMI	− 0.220	− 0.340	0.737			0.0	− 0.049	0.962			0.046	0.185	0.855			− 0.128	− 0.469	0.644		
EDI-2—body dissatisfaction	**0.582**	**2.030**	**0.054**			**0.3**	**2.323**	**0.029**			− 0.054	− 0.485	0.632			0.118	0.977	0.339		
EDI-2—interoceptive awareness	0.248	0.776	0.446			0.0	0.163	0.872			0.060	0.482	0.634			0.092	0.682	0.502		

	CADSS tot					CADSS der					CADSS dep					CADSS D.A				
	*b*	*t*	*p*	*R*^2^	Adj *R*^2^	*b*	*t*	*p*	*R*^2^	Adj *R*^2^	*b*	*t*	*p*	*R*^2^	Adj *R*^2^	*b*	*t*	*p*	*R*^2^	Adj *R*^2^
Intercept	13.204	0.774	0.447	0.573	0.499	9.407	0.876	0.390	0.445	0.349	3.475	0.543	0.592	0.579	0.505	0.322	0.117	0.908	0.554	0.477
Group	0.046	0.006	0.996			0.100	0.019	0.985			1.449	0.473	0.641			− 1.503	− 1.137	0.267		
BMI	− 0.272	− 0.364	0.719			− 0.188	− 0.399	0.693			− 0.094	− 0.336	0.740			0.010	0.080	0.937		
EDI-2—body Dissatisfaction	0.499	1.505	0.146			0.312	1.499	0.148			0.067	0.537	0.596			**0.119**	**2.228**	**0.036**		
EDI-2—interoceptive awareness	**0.892**	**2.415**	**0.024**			0.325	1.397	0.176			**0.370**	**2.674**	**0.014**			**0.197**	**3.297**	**0.003**		

*Adj R*^*2*^ Adjusted R^2^, *BMI* Body Mass Index, *CADSS* Clinician-Administered Dissociative States Scale, *Der* Derealization, *Dep* Depersonalization, *D.I.* Dissociative Identity, *D.A.* Dissociative Amnesia, *EDI-2* Eating Disorder Inventory – 2, *SFQ* Strange Face Questionnaire, *Tot* Total Score

Bold values are significant values (*p* < 0.05)

## Discussion

The aim of the present study was to assess dissociative symptoms elicited by mirror exposure in a particular condition (sensory deprivation due to diminished illumination) through the MGT, in a group of patients with AN and a group of HC. Novel aspects of this study are mainly two: first, dissociation was evaluated both through a specific task and specific questionnaires (differently from previous studies, where only questionnaires were used); second, the instrument used here to trigger dissociative features involved the presence of a mirror, which is known to elicit body image disruption in patients with AN. Concerning this last point, it is worthwhile mentioning that here we compared the participants only with the mirror image of their own face, while in previous cited studies participants viewed their full body in the mirror [6–9]. Although it is questionable whether this confrontation will elicit the same signs of body image disturbance like viewing the full body in the mirror, this is the first study using this kind of instrument to evoke dissociation. Concerning this point, in the MGT, it is expected that the participant stares directly at his/her own eyes reflected in the mirror, although apparitions can be elicited even staring at other points on the face (i.e., the forehead) [26]. Manipulations of the MGT have been performed by asking couples of participants to stare into each other’s eyes, and strange-face-illusions emerged as well [34]. The face is a complex stimulus pattern, compared to the body, and moreover, it conveys a complete self-identity representation. Thus, the face, which is reflected in the mirror or perceived through the other's gaze, is the ideal stimulus to investigate three different facets of dissociation (derealization, depersonalization, and dissociative identity), as it was showed in a previous study [27]. Here, we decided to perform the MGT in its original version but, given the body image concern present in patients with AN, it will be arguably of interest to investigate the presence of illusions and dissociation even in a full-body MGT.

Our data showed a strong dissociation pattern for the AN group, contrary to the HC group. AN patients, in fact, presented a higher number of misperceptions by looking in the mirror, as assessed via the SFQ, and scored higher both at the subscale Dissociative Identity/Compartmentalization of the SFQ and at the CADSS total score and at all its subscales (Derealisation, Depersonalization, Dissociative Amnesia). It must be noted that SFQ items associated to derealisation are specifically linked to visual perceptual deformations of facial features, and these anomalous visual perceptions resulted similar between AN and HC; on the other hand, CADSS derealisation items are generally linked to colour changes, tunnel vision, and anomalous auditory perceptions, and these resulted different between AN and HC.

As anticipated in the introduction, it has been hypothesized that strange-face apparitions might emerge during MGT because of a disconnection of two facets of representations that are usually recruited together and allow self-recognition in the mirror: the one’s own embodied self-representation and the visual representation of one’s own face left–right reversed, as it usually appears in the mirror. According to data available in the literature, we might speculate that this disconnection is reached at different times by different groups of patients, in a way that is strongly linked to their own psychopathology: schizophrenic patients did not only dissociate their identity, but also identified themselves with the apparitions in the mirror, contrary to healthy individuals [29]; depressed patients, on the contrary, experienced a lower number of apparitions, probably because of their deficits in emotional facial recognition and expression [31]. Here, due to body image distortion (directly stimulated by looking in the mirror) [6–13] and the tendency to experience dissociation of patients with AN [17–21], we might think that they reach the threshold for a disconnection of the two over mentioned facets of representation before HC. This hypothesis is just speculative, since it was not directly examined in the present study, and therefore, it is difficult to conclude whether different results might be due to differences in timing (e.g., onset of the first anomalous experience in MGT, which was found to be much reduced in schizophrenic patients [29]) or some other aspects (e.g., the prodromal role of body image distortion on dissociative detachment of bodily-self); hence, we would suggest to conduct future studies to specifically examine this question. However, our hypothesis, although speculative, is further sustained by a recent study of our research group showing that patients with functional neurological disorders, a neuropsychiatric condition which shares several psychopathological features with AN [39], presented higher levels of dissociation than HC after the MGT exposure [30]. However, when controlling for other variables such as the BMI and the selected EDI-2 subscales, the variable Group was not significantly associated neither to the SFQ nor to the CADSS, suggesting that the reason for the differences between the two groups should be found in other specific psychological features. Similarly, the BMI did not correlate with any of the MGT variables: this result is apparently in contrast with previous research findings according to which AN symptoms can be exacerbated by being underweight or in a malnourished state [40]; here, we might hypothesize that dissociative symptoms follow a different pattern, which is independent from BMI.

In this study, although we assessed only one facet of body image dysfunction (body dissatisfaction), we showed that the Body Dissatisfaction subscale of the EDI-2 was correlated to the SFQ subscale Derealisation and to the CADSS subscale Dissociative Amnesia. Moreover, a trend towards significance emerged for the relationship between the subscale Body Dissatisfaction and the Total Score of the SFQ. This result confirms the hypothesis according to which dissociation and body image dysfunction are strongly linked in the pathophysiology of AN [17–20]. Our finding might also have some therapeutic implications, especially in psychotherapy setting, where techniques focusing on the treatment of dissociative symptoms should be encouraged. Concerning this aspect, the use of dissociation–induction procedures, combined with other therapeutic techniques, such as grounding, emotion- or soma-focused attention training, should be considered as therapeutic tools to facilitate identification and management of dissociative symptoms in patients with AN [41]. As discussed above, body image disruption is a key pathophysiological feature also of Body Dysmorphic Disorder, a disorder characterized by the obsessive idea that some aspects of one's own body part or appearance are severely flawed, and therefore, warrant exceptional measures to hide or fix one’s dysmorphic part on one’s figure. Recently some authors proposed a new diagnostic category that encompasses both diseases: the so-called “Body Image Disorders” [32]. BDD patients usually spend many hours in front of a mirror [43–46], where they perform “mental cosmetic surgery” to change their body image [47]. It has been speculated that BDD patients may achieve apparitional experiences and dissociation of identity during mirror gazing because of their pathological habit to modify their own body image in front of a mirror: the appearance of a strange, difference face could be felt as the result of the whole package of “safety behaviours” performed in front of a mirror [47], with the consequence of promoting a new dissociative identity [26]. Our data shows that this hypothesis should be extended to patients with AN as well. Dissociative identity (formerly called multiple-personality [48]) was triggered by MGT, hence indicating strong compartmentalization [49] of two or more identities in AN patients. The advantage of MGT as a therapeutic tool may be that patients can bring consciousness on their alter-identities, which otherwise would remain unconsciously compartmentalized.

Finally, we found that the EDI-2 subscale Interoceptive Awareness was a correlated to the CADSS Total Score and to the CADSS subscales Depersonalization and Dissociative Amnesia scores; more specifically, the higher the difficulties in perceiving one’s own body inner states, the higher the tendency to dissociation. Given that, in our sample, body dissatisfaction and disruption in interoceptive awareness were both correlated to dissociation, we might speculate that our data also support the hypothesis of a link between body image distortion in patients with AN and an abnormal functioning of the insula, which integrates interoceptive sensations [14, 15]. A recent study showed that mirror gazing can positively enhance aspects of self-processing: interoceptive accuracy, measured with a Heartbeat Detection Task, increased in healthy participants when they performed the task while directly looking at one’s own face in a mirror [50]. Given that it is well-established that AN patients have lower interoceptive accuracy than HC [51], mirror gazing condition might be optimal to increase body awareness in this population. This hypothesis might represent a possible neurobiological explanation for the frequent use of mirror exposure therapy (a clinical trial validated treatment component that improves body image and body satisfaction in ED [52, 53]) during the treatment of patients with AN in psychotherapy setting.

Our study has the following limitations: first, the small sample size might limit the generalization of our findings and the reliability of statistical analysis, although restrictive non-parametric tests have been used and the limit of “two subjects per variable” in the multiple linear regression has been respected [38]; second, since men and women differ in their body image disturbances and in their exposure to traumatic events [54, 55], an all-female control group would have been preferable; however, in our sample, sex-differences were not significant between the groups and when running the analyses without male participants, results remained the same; third, we assessed only one facet of body image disruption (body image dissatisfaction), but we did not assess perceptual distortion; fourth, although the instrument used (MGT) is gaining more and more evidences of reliability in different clinical populations, our data would be stronger if confirmed by other experimental paradigms aimed to assess the process of dissociation; fifth, reliability for the SFQ subscale Depersonalisation and the CADSS subscale dissociative amnesia were low; however, future studies focusing on the questionnaires’ validation will take this point into account; sixth, here participants, differently from previous studies [29], after the MGT were not first interviewed with open questions about their experiences, but immediately answered Likert-type questions about their experiences, which might be more prone to suggestibility; seventh, we used the EDI-2 to assess eating disorder psychopathology and not the last version of the EDI (EDI-3); eight, we excluded patients with other comorbid psychiatric conditions to have a more homogeneous group; however, this might represent a bias of our results, since other psychiatric comorbidities are common in AN. Finally, since we did not consider pre-mirror exposure dissociation symptoms or compare AN groups with and without mirror exposure on dissociative symptoms, we cannot interpret whether the symptoms were elicited or altered by the task, as opposed to being pre-existing.

In conclusion, our study showed that patients with AN presented more dissociative symptoms than HC, when assessed through the MGT, a specific objective instrument. In addition, the increase of dissociative symptoms was correlated with body dissatisfaction and disruption in interoceptive awareness. This study not only provides additional knowledge on the pathophysiology of AN, exploring dissociative symptoms with a novel instrument, but also has important clinical and therapeutic implications, providing further evidence for the use of techniques focusing on the treatment of dissociative symptoms and for the use of mirror exposure therapy during the treatment of patients with AN in psychotherapy setting. For this reason, it might be of interest to implement the MGT in a full-body session, where the participants are allowed to see their whole body reflected in the mirror.

Future researches in larger populations should expand our preliminary results. Moreover, replicating our results in patients affected by BDD would give an additional evidence to our findings.

### What is already known on this subject?

Previous studies on patients with anorexia nervosa underlined a strong linked between dissociative symptoms and body image disruption. However, one of the main limitations of these previous studies was the lack of an objective assessment of dissociation and its subcomponents, which were evaluated only through self-report scales and not by experimental tasks.

### What this study adds?

Our study showed that patients with anorexia nervosa presented more dissociative symptoms than healthy controls, when assessed through the mirror gazing test, a specific objective instrument. In addition, the increase of dissociative symptoms was correlated with body dissatisfaction and disruption in interoceptive awareness. These findings might also have some important therapeutic implications.

## Data Availability

Anonymized data will be shared by request from any qualified investigator.
